# Advancing Primary Care for Older Adults Through the 4M Framework and Resource Exchange

**DOI:** 10.1093/geroni/igaf122.3150

**Published:** 2025-12-31

**Authors:** Victoria O’Connor, Christine Ferrone, Susanne Campbell, Suzanne Herzberg, Nijah Venditelli, Jayne Daylor

**Affiliations:** Rhode Island Department of Health, Providence, Rhode Island, United States; University of Rhode Island, Kingston, Rhode Island, United States; Care Transformation Collaborative of Rhode Island, Providence, Rhode Island, United States; Care Transformation Collaborative of Rhode Island, Providence, Rhode Island, United States; Care Transformation Collaborative of Rhode Island, Providence, Rhode Island, United States; Care Transformation Collaborative of Rhode Island, Providence, Rhode Island, United States

## Abstract

The growing population of adults aged 65+ will strain primary care, increasing demand on a limited workforce. The Age-Friendly 4M framework—Medication, Mentation, Mobility, and What Matters—offers a structured, evidence-based approach to team-based primary care. This project used a resource-exchange strategy to support 4M adoption in primary care. The Rhode Island Department of Health Alzheimer’s Disease and Related Disorders “BOLD” Program, the Care Transformation Collaborative of RI, the RI Geriatrics Workforce Enhancement Program, and UnitedHealthcare collaborated to launch two six-month quality improvement initiatives to assist primary care practices with implementing the 4M framework and achieving the Institute for Healthcare Improvement’s (IHI) Age-Friendly recognition. Identification of needed resources and support for what matters most to care partners of patients with dementia were key added components. Practices responded to a call for applications and received funding, individualized support from a practice facilitator, and structured workplans to engage at Level 1 (preparing for 4Ms) or Level 2 (expanding 4M processes). Practices met with facilitators monthly and joined three peer learning sessions. Over two years, 11 practices participated. All practices achieved IHI Level 1 designation, and all five that applied advanced to Level 2. Pre- and post-program ratings showed a 22% overall improvement in adherence to 16 distinct 4M guidelines and dementia care partner engagement. By pooling resources, this initiative advanced 4M adoption in primary care, enhancing care for older adults and dementia patients by identifying care partners, addressing their needs, and offering community support. Partner collaboration ensured engagement and sustainability.

